# Rapid switching and durable on-chip spark-cavitation-bubble cell sorter

**DOI:** 10.1038/s41378-022-00382-2

**Published:** 2022-05-18

**Authors:** Zeheng Jiao, Yong Han, Jingjing Zhao, Zixi Chao, Attila Tárnok, Zheng You

**Affiliations:** 1 State Key Laboratory of Precision Measurement Technology and Instrument, Tsinghua University, Beijing, 100084 China; 2 Department of Precision Instrument, Tsinghua University, Beijing, 100084 China; 3 Beijing Laboratory for Biomedical Detection Technology and Instrument, Tsinghua University, Beijing, 100084 China; 4 Department of Structural Biology, Stanford University, School of Medicine, Stanford, CA 94305-5126 USA; 5 Institute for Medical Informatics, Statistics and Epidemiology (IMISE), University of Leipzig, Leipzig, Germany; 6 Department of Therapy Validation, Fraunhofer Institute for Cell Therapy and Immunology IZI, Leipzig, Germany

**Keywords:** Microfluidics, Electrical and electronic engineering

## Abstract

Precise and high-speed sorting of individual target cells from heterogeneous populations plays an imperative role in cell research. Although the conventional fluorescence-activated cell sorter (FACS) is capable of rapid and accurate cell sorting, it occupies a large volume of the instrument and inherently brings in aerosol generation as well as cross-contamination among samples. The sorting completed in a fully enclosed and disposable microfluidic chip has the potential to eliminate the above concerns. However, current microfluidic cell sorters are hindered by the high complexities of the fabrication procedure and the off-chip setup. In this paper, a spark-cavitation-bubble-based fluorescence-activated cell sorter is developed to perform fast and accurate sorting in a microfluidic chip. It features a simple structure and an easy operation. This microfluidic sorter comprises a positive electrode of platinum and a negative electrode of tungsten, which are placed on the side of the main channel. By applying a high-voltage discharge on the pair of electrodes, a single spark cavitation bubble is created to deflect the target particle into the downstream collection channel. The sorter has a short switching time of 150 μs and a long lifespan of more than 100 million workable actions. In addition, a novel control strategy is proposed to dynamically adjust the discharge time to stabilize the size of the cavitation bubble for continuous sorting. The dynamic control of continuously triggering the sorter, the optimal delay time between fluorescence detection and cell sorting, and a theoretical model to predict the ideal sorting recovery and purity are studied to improve and evaluate the sorter performance. The experiments demonstrate that the sorting rate of target particles achieves 1200 eps, the total analysis throughput is up to 10,000 eps, the particles sorted at 4000 eps exhibit a purity greater than 80% and a recovery rate greater than 90%, and the sorting effect on the viability of HeLa cells is negligible.

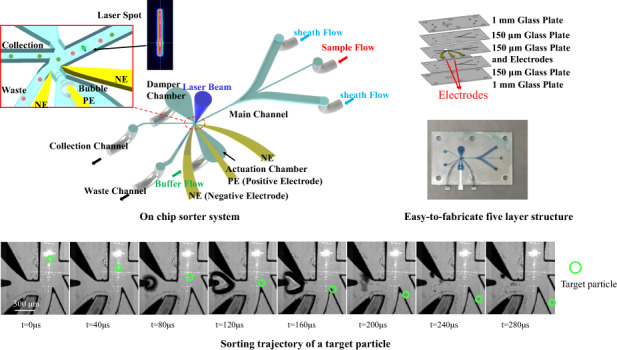

## Introduction

Fluorescence-activated cell sorting (FACS) is a widely used tool in biomedical research and cell diagnostics. In FACS, individual cells are encapsulated in electrically charged droplets and then electrostatically deflected into different tubes in an open space. This jet-in-air sorting method is highly efficient, but it will induce aerosol exposure risk for the operator and cross contamination between adjacent samples. Sorting in fully enclosed and disposable microfluidic chips can address these limitations. Various mechanisms to achieve microfluidic-based fluorescence-activated cell sorting (μFACS) have been demonstrated in the past two decades. Generally, early-stage methods have low switching efficiency (typically >10 ms) and low throughput (typically <100 eps), including electroosmosis^1,2^, electrowetting^3^, dielectrophoresis^4,5^, opto-caloric^6^, and optical tweezers^7,8^. Microfluidic-based droplet sorters allow a sorting throughput higher than 2000 droplets/s^9,10^, but their two-phase liquid–liquid system (aqueous droplets in oil medium) complicates the fluidic system and requires an additional cell recovery process. Sorters based on acoustic waves^11,12^, piezoelectric actuator (PZT) membranes^13–15^ and MEMS actuators^16^ can achieve a high sorting rate (>2000 target cells/s), but they require precise assembling and integrating of PZT or interdigital transducer (IDT) onto the microfluidic chip. The lifetimes of most sorters are limited to a few million sort events due to thermal and mechanical damage^17^, especially for those owning moving parts^18^.

Microbubble-based cell sorting is a recently emerging method. It applies an expanding microbubble to generate a jet flow that can deflect the target cell, showing a rapid switching time on the scale of 100 μs and a high cell viability. Pulsed laser^19–21^ and resistive heating^22,23^ are the two major approaches to generating microbubbles, but their systems are complex. In the laser-induced bubble sorting system, a high-power picosecond or nanosecond pulsed laser should be accurately focused on a specific spot in the microfluidic chip. For the resistive-heating-generated vapor bubble sorter, MEMS fabrication and assembly are required to integrate micro heaters into the microfluidic chip. Spark discharge^24,25^ is another method for the generation of microbubbles. Our previous work^26^ has demonstrated the possibility of using spark cavitation bubbles for on-chip sorting. It applies a high-voltage pulsed discharge on a pair of electrodes in the microfluidic chip for the electrical breakdown of the aqueous solution, resulting in a single cavitation bubble. The previous design cannot make continuous sorting as the microfluidic structures and system control have not been optimized, leading to a short lifetime (10^5^ sort events) and a long switching time (250 μs).

Here, we propose a high-speed and durable spark-generated bubble-based microfluidic cell sorter. Compared to our previous work, the sorter is optimized to shorten the switching time down to 150 μs, increasing the maximum sorting throughput. In addition, platinum and tungsten are selected as the electrode materials for the positive electrode and negative electrode, which prolongs the sorter lifetime to more than 10^8^ operations. Third, an algorithm to dynamically control the spark cavitation is developed to maintain the sorting stability and accuracy. These improvements make the on-chip spark-cavitation-bubble cell sorter work continuously and precisely. At a throughput of 4000 events per second, the sorting purity is better than 80%, and the recovery rate is better than 90%. The outstanding advantage of this sorter is the simplicities of its structure and setup. The sorting is easily realized by a pair of electrodes and requires no complicated off-chip setup.

## Sorting principle

**Fig. 1:**
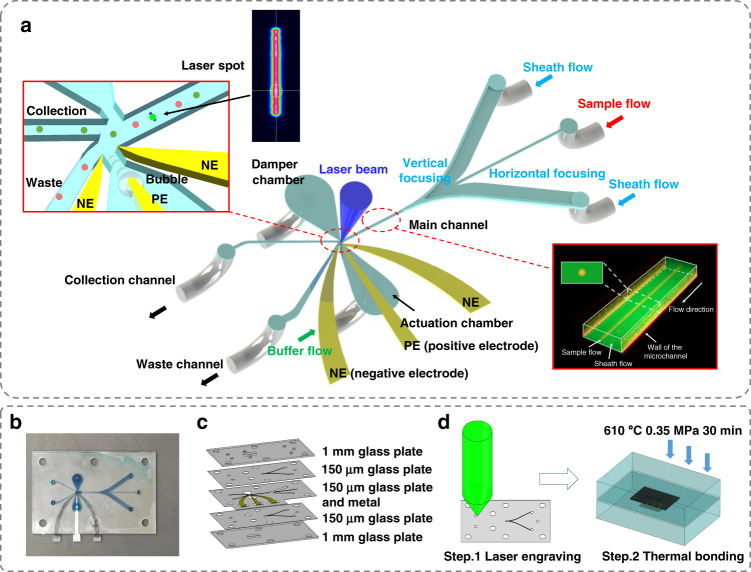
The spark-generated bubble-based on-chip cell sorter. **a** Schematic of the chip. The sorting region, flat-top laser spot, and 3D hydrodynamic focusing of the sample flow are shown in the insets. **b** Photo of the microfluidic chip. **c** The five-layer structure of the chip. **d** The two-step fabrication procedure. PE positive electrode, NE negative electrode.

The design of the cell sorter is depicted in Fig. 1a, b. The microfluidic chip consists of five glass plates, as shown in Fig. 1c. The three inner plates are 150 μm thick, where the microfluidic channels complete the 3D hydrodynamic focusing of the sample flow^27^. The third layer is composed of two upstream sheath-flow channels for horizontal hydrodynamic focusing, a straight main channel, a waste channel, a collection channel, and a pair of electrodes. The main channel is connected to the downstream collection channel and waste channel via a bifurcating junction. The thin needle-like structure and arc-shaped parts are positive electrodes (PE) and negative electrodes (NEs), respectively. The actuation chamber is linked to the main channel through a nozzle that is close to the bifurcating junction. The second and fourth glass layers contain microchannels for vertical hydrodynamic focusing. The two outer 1-mm glass plates cover the microfluidic chip and offer an observation window and fluid connection ports.

The flowing particles are successively illuminated by a rectangular flat-top 488-nm laser beam, which is shaped by a diffractive optical element (DOE)^28^, as shown in Fig. 1a. If a target particle is detected, a high-voltage electric discharge will be applied to the electrodes after a certain delay, generating a dielectric breakdown of the solution between the electrodes. The deposited energy leads to localized heat and initiates a cavitation bubble^29^. The bubble expands, drives the surrounding liquids, and gives a jet flow through the nozzle into the main channel. As a result, the target particle is deflected by the jet flow and flows into the collection channel. Once the maximum volume is reached, the microbubble will rapidly shrink and collapse. Then, the liquids recover to insulation, and the sample flows back into the waste channel. The electric discharge is confined in the actuation chamber to prevent electric field shock from damaging the cells in the main channel (Fig. S1 shows the simulated electric field). The flow rate of the sheath flow is 120 μL/s and that of the sample flow is 0.5–2 μL/s. The sheath flow confines the sample flow to be a narrow stream with a diameter of 15 μm and a velocity of 5 m/s. This high-quality 3D hydrodynamic focusing is fundamental for accurate sorting. We applied buffer flow (40 μL/s) to refresh the liquids between the electrodes and sweep away electrolytic microbubbles^30^ that are generated simultaneously with the cavitation bubble. Sheath and buffer liquids are 1× phosphate buffer saline (PBS), which can maintain the osmolality of the cells and reduce the threshold voltage for bubble generation^31^.

**Fig. 2:**
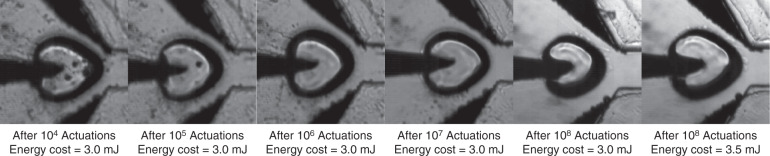
Photos of the cavitation bubbles after different times of spark discharges. The picture 1-5 from left to right shows bubbles after 10^4^~10^8^ cavitation actuations. After 10^8^ actuations, the bubble size will slightly decrease. Picture 5-6 shows that the bubble size could be reinstated by increase the energy cost.

The glass plates and metal electrodes are fabricated by laser engraving and integrated using thermocompression bonding^32^, as illustrated in Fig. 1d. The chip in this work has the same five-layer structure as our previous work^32^, except for inserting three metal electrodes in the third layer (Fig. S2). The fabrication details are listed in a previous work^32^, including laser processing, glass cleaning, chip alignment, thermocompression bonding, and cooling. One chip is produced within 5 h and costs no more than 20 USD. The low cost makes the chip disposable to eliminate cross contamination. Platinum and tungsten are selected as the materials for PE and NEs, respectively. The gap between PE and NEs is determined via experiments (see Fig. S3 and the explanation). Figure 2 proves that the lifetime of the electrodes is more than 10^8^ spark discharges. After 10^7^ actions, the electrodes remained intact with no degeneration. After 10^8^ actions, the cavitation bubble decreases because of the spark-induced erosion on the PE tip. The bubble size can be reinstated by slightly increasing the discharge energy from 3.0 to 3.5 mJ. In other words, the sorter is still workable after 10^8^ actions. Stainless-steel PE is used in our previous design^26^ with a lifetime less than 10^5^, as shown in Fig. S4.

### System setup

**Fig. 3:**
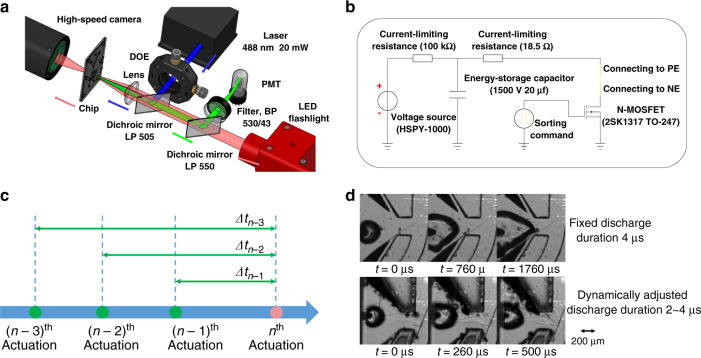
The setup of the cell sorter. **a** Laser illumination and fluorescence detection. **b** The high-voltage circuit is used to trigger the spark cavitation bubble. The discharge is controlled by the N-MOSFET. **c** The algorithm adjusts the discharge duration according to the time intervals of the former three spark discharges. **d** The bubble size increases gradually if the discharge duration is constant. With the dynamically adjusted discharge duration, the bubble size is steadier even with shorter time intervals.

The optical detection of the cell sorter is shown in Fig. 3a. A 488-nm laser (20 mW) is shaped by a diffractive optical element^28^ (DOE) and focused through a lens (*f* = 40 mm) to form a rectangular flat-top spot (10 μm × 60 μm). The emitted fluorescence of each particle is collected by the same lens, extracted by two dichroic mirrors (longpass 505 nm and longpass 550 nm) and a bandpass filter (530 nm/43 nm), and then detected using a photomultiplier tube (PMT, R928 + C7427, Hamamatsu, Japan). A sorting command will be given to the high voltage circuit with a certain delay to trigger a spark cavitation bubble if the PMT signal of the fluorescence surpasses the threshold value (Fig. S5). With the LED flashlight, a high-speed camera (Photron FastCam SA-Z, Photron Inc., Japan) is installed at the opposite side of the chip to capture the sorting process. Three syringe pumps (TS-1B, LongerPump, China) drive the sample flow, the sheath flow, and the buffer flow. The high-voltage circuit shown in Fig. 3b generates individual spark discharges. A 900-V electric discharge with a duration between 2 and 4 μs is applied to the electrodes when a sorting command is received.

During the sorting operation, spark cavitation bubbles are produced repeatedly with variable time intervals. Since spark discharges increase the conductivity of aqueous solution, although we use buffer flow to refresh the cavitation region, the successive cavitation bubble will enlarge if the time interval between two adjacent discharges is too short^31^. This will lower the sorting stability and accuracy. To keep the repeated cavitations stable, we developed an algorithm to dynamically adjust the discharge duration. According to our previous research, there is a linear relationship between the volume of the cavitation bubble and the discharge duration^31^. When the instantaneous activation frequency increases, the discharge duration will be shortened to reduce the energy deposited on the electrodes. Consequently, it can neutralize the influence of the increased conductivity of the aqueous solution, and the bubble size is kept steady during the high-frequency sorting period. The duration of the *n*th action is adjusted according to its time intervals to the previous three actions, as expressed below.

1 $$\begin {array}{l}t_n = t_{st}\left[ {1 - k_1\left( {1 - \frac{{\Delta t_{n - 1}}}{{\Delta t_{st}}}} \right) \cdot H\left( {1 - \frac{{\Delta t_{n - 1}}}{{\Delta t_{st}}}} \right) - k_2\left( {2 - \frac{{\Delta t_{n - 2}}}{{\Delta t_{st}}}} \right)}\right.\\\qquad \left.{\cdot H\left( {2 - \frac{{\Delta t_{n - 2}}}{{\Delta t_{st}}}} \right) - k_3\left( {3 - \frac{{\Delta t_{n - 3}}}{{\Delta t_{st}}}} \right) \cdot H\left( {3 - \frac{{\Delta t_{n - 3}}}{{\Delta t_{st}}}} \right)} \right]\end {array}$$

2 $$H\left( x \right) = \left\{ {\begin{array}{*{20}{c}} 1 & {x \,\ge\, 0} \\ 0 & {x \,<\, 0} \end{array}} \right.$$

where *t*_*n*_ is the discharge duration of the *n*th sorting operation, *t*_*st*_ = 4 μs is the standard discharge duration, and $\Delta t_{st} = 500\upmu {\rm{s}}$ is the standard time interval between two adjacent actions. $\Delta t_{n - 1}$, $\Delta t_{n - 2}$ and $\Delta t_{n - 3}$ are the time intervals between the current action and the three previous actions. *k*_1_, *k*_2_ and *k*_3_ are the adjustment coefficients, which are determined to be 0.30, 0.10, and 0.05 in our experiments. By applying the dynamic adjusting algorithm, the bubble size is significantly stabilized (Fig. S6 shows the stabilizing effect of the algorithm).

### Sample preparation

Green (Ex 470 nm/Em 526 nm) and red (Ex 620 nm/Em 680 nm) fluorescent polystyrene beads of different sizes (BeasLine Tech, China) were employed to calibrate the sorter and characterize its sorting capacity. HeLa cells were used to evaluate the sorting impact on cell viability. Green fluorescent protein (GFP)-expressing HeLa cells and red fluorescent protein (RFP) HeLa cells were cultured in RPMI Medium Modified (HyClone, SH30809.01) with 10% fetal bovine serum (Thermo Fisher, 10091148) and 1% penicillin–streptomycin (all from HyClone, SV30010) in a cell incubator (HF90, HealForce, China) at 37 °C with 5% CO_2_. Prior to use, the HeLa cells were detached from the flask with trypsin (0.25%, HyClone), washed and resuspended in a mixture of PBS and OptiPrep density gradient medium (D1556, Sigma–Aldrich, St. Louis, MO) in an 88:12 volume ratio. Concentrations are adjusted to ∼10^6^ beads per mL for the polystyrene beads and ∼5 × 10^6^ cells per mL for the HeLa cells. To measure viability, HeLa cells were incubated on ice with PI (propidium iodide) solution (500 μg/mL, RuiTaiBio, China) at 4 μL/mL for 5 min. Dead cells stain positively, and thus, viability can be measured by counting the percentage of cells that stain negatively.

## Results and discussion

### Sorting process

**Fig. 4:**
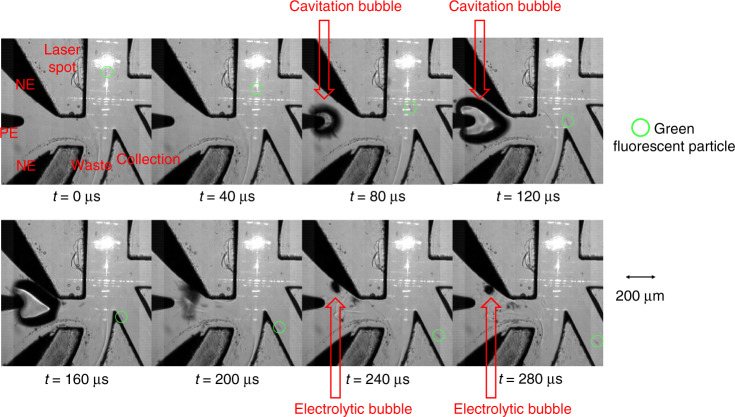
Sorting process of a single 10-μm green fluorescent bead. Green circle shows the position of the bead during 0~280 μs, the caviation bubble and electrolytic bubble are indicated by red arrows.

Figure 4 shows the sorting process of a single 10-μm green fluorescent bead. The delay time between the fluorescence signal and the spark discharge is set at 80 μs (refer to the sorting envelope section). The lifetime of the spark cavitation bubble is 150 μs with an almost symmetric expansion phase and collapse phase. The sorting of three microbeads passing successively with a time interval of 600 μs is shown in Fig. S7 and supplementary video.

### Sorting envelope

The sorting envelope is defined as a range of delay times^17^, in which almost 100% of the target particles are successfully sorted. The false-positive error rate will increase if the sorting envelope is too long, and the false-negative error rate might increase if it is too short. We generated six different-sized bubbles by varying the discharge duration (Table 1) and determined the sorting envelope of each bubble by sweeping the delay time with a step of 10 μs. The six bubbles in Table 1 were selected to cover a wide range of sizes in the actuation chamber (Supplementary Fig. S11). The bubble volume is derived by the product of the projected area and actuation chamber height.

**Table 1:** Six different sized bubbles were generated in the experiment.

	Bubble A	Bubble B	Bubble C	Bubble D	Bubble E	Bubble F
Energy cost (mJ)	2.24	2.68	3.05	3.49	3.95	4.43
Volume (μm^3^)	6.2 × 10^6^	8.4 × 10^6^	12.2 × 10^6^	14.4 × 10^6^	20.5 × 10^6^	23.4 × 10^6^

**Fig. 5:**
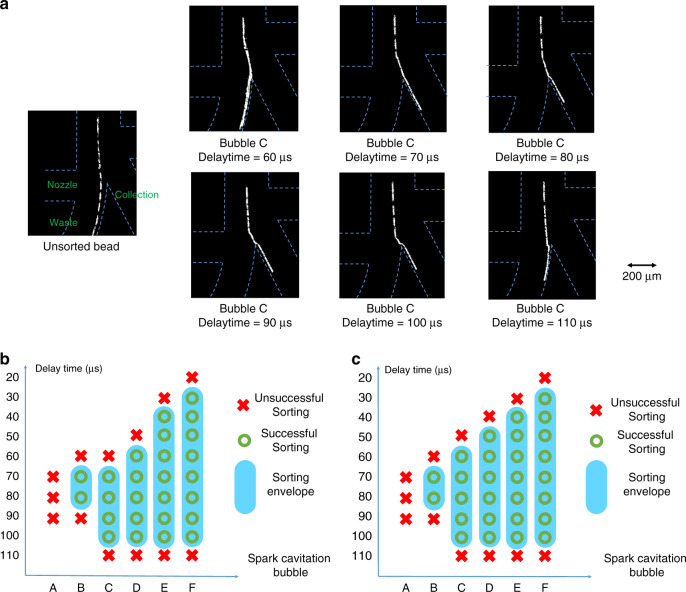
The sorting envelope under various conditions. **a** Trajectories of unsorted beads and beads deflected by bubble C with different delay times. **b** Sorting envelope for 10-μm polystyrene beads. **c** Sorting envelope for 5-μm polystyrene beads.

Taking bubble C as an example, Fig. 5a shows the trajectories of an unsorted bead and the beads deflected by bubble C with disparate delay times. The sample was a 10-μm green polystyrene bead. When the delay time is 60 μs, the bead is pushed by the jet flow but then pulled back to the waste channel due to the reverse jet flow induced by the shrinking of the cavitation bubble. For the delay time of 110 μs, the generation of the cavitation bubble is so late that the bead is blocked by the junction and ends up in the waste channel. The sorting envelope of bubble C is estimated to be 40 μs. The sorting envelopes of the different sized bubbles for 10-μm fluorescent beads and 5-μm fluorescent beads are described in Fig. 5b, c, respectively. Bubble C is used for the later experiments, considering its relatively long sorting envelope and low energy cost (small size). Additionally, the delay time is set to 80 μs, which is in the sorting envelope of bubbles B~D and is available for a wide range of bubble sizes.

### Theoretical purity and recovery rate

Purity is calculated as the percentage of sorted target particles out of the total sorted particles. The recovery rate represents the percentage of sorted target particles in the total target particles. The time distribution of the particles aligned in the focused sample flow entering the sorting region is random. The time intervals between two successive particles follow a Poisson distribution, expressed as $P\left( {T < t} \right) = 1 - {{{\mathrm{e}}}}^{ - Nt}$^17,33^, where *N* is the total throughput. Figure S8 proves that the experimental result is coincident with the Poisson distribution. We can calculate the theoretical purity and recovery rate under two assumptions (this model is also referred to as the theoretical ideal sorter). First, false-positive error (nontarget particles in collection channel) only occurs when a nontarget particle is in the sorting envelope of a target particle by coincidence. Second, false-negative error (target particles in the waste channel) only occurs when the time interval of two adjacent target particles is shorter than the minimum repeat time of the sorter; thus, the sorter will not trigger a second spark discharge for the second target particle. The minimum repeat time *H*_1_ of this sorter is chosen as 200 μs. If the sorting event is set in the middle of the sorting envelope, the false negative error rate and the false-positive error rate are calculated as follows:

3 $$P_{fn} = P\left( {T_{ta} \,<\, H_1} \right) = 1 - e^{ - N_{ta}H_1}$$

4 $$P_{fp} = 2P\left( {T \,<\, \frac{{H_2}}{2}} \right)\frac{{N - N_{ta}}}{N} = \left( {1 - {{{\mathrm{e}}}}^{ - \frac{{NH_2}}{2}}} \right)\frac{{2\left( {N - N_{ta}} \right)}}{N}$$

where *T*_*ta*_ is the time interval between two successive target particles, *H*_2_ is the sorting envelope of the nontarget particle, and *N*_*ta*_ is the throughput of the target particles. For example, if the sample is a mixture of 10-μm green polystyrene target beads and 5-μm red fluorescent nontarget beads, $H_2 = 50\upmu {\rm{s}}$, $N_{ta} = 500$ and $N = 3000$ then the false negative error rate *P*_fn_ is 9.5% and the false-positive error rate *P*_fp_ is 12.0%.

### Beads sorting

The purity and recovery rate of bead sorting experiments are investigated at various throughputs. The sorter works in enrich mode, which means it sorts with no rejection to the positive particles, except if the time interval is shorter, the minimum actuation repeat time. The sorting results of this microfluidic sorter were examined by a standard flow cytometer (BD LSR Fortessa, BD Biosciences).

The first sample only contains pure 10-μm green fluorescence target beads, with the results listed in Table 2. The purpose is to test the continuous sorting performance and evaluate the false negative error rate. The recovery rate decreases with increasing target bead throughput. The experimental recovery rate is approximately 80% at the target beads throughput of 1000 eps. When the target bead throughput is higher (>500 eps), the frequent cavitation makes it difficult for the flows to fully recover before every sorting event. The stability of the sorting system is influenced, and the experimental recovery rate is slightly lower than the theoretical rate.

**Table 2:** Sorting of pure green PS target beads

Test	Target beads throughput (eps)	Theoretical recovery rate	Experimental recovery rate
1	286	94.4%	94.7%
2	495	89.3%	90.3%
3	893	83.8%	80.8%
4	1213	78.4%	75.7%

The second sample is the mixture of 10-μm green fluorescent target beads and 5-μm red fluorescent nontarget beads, and the mixture has a low percentage of the target beads. This experiment mimics the enrichment of very rare cell samples. As shown in Table 3, the experimental purity is slightly lower than the theoretical purity mainly for two reasons. First, a small number of nontarget beads may directly enter the collection channel due to hydrodynamic focusing fluctuation. Second, although modulated, the volume of the cavitation bubble is not perfectly maintained. The increase in bubble volume in the long-term sorting operation results in the variation of the sorting envelope.

**Table 3:** Rare sorting experiments.

Test				Theoretical		Experimental	
	Target beads throughput (eps)	Total beads throughput (eps)	Presorting target beads fraction	Recovery rate	Purity	Recovery rate	Purity
1	357	9921	3.6%	93.1%	70.3%	95.0%	61.5%
2	318	10,818	2.9%	93.9%	68.5%	95.1%	55.9%
3	241	19,326	1.2%	95.3%	56.9%	94.3%	47.0%
4	379	32,297	1.2%	92.7%	47.7%	92.7%	40.7%

The third kind of sample is also a mixture of 10-μm green fluorescent target beads and 5-μm red fluorescent nontarget beads, but the percentages of the target beads and the nontarget beads are comparable. Table 4 shows the experimental results. The experimental recovery rate is close to the theoretical results. The experimental purity is lower than the theoretical purity for similar reasons in the rare sorting experiments.

**Table 4:** Common sorting experiments

Test				Theoretical		Experimental	
	Target beads throughput (eps)	Total beads throughput (eps)	Presorting target bead fraction	Recovery rate	Purity	Recovery rate	Purity
1^a^	102	715	14.3%	98.0%	97.1%	98.3%	93.6%
2^a^	251	1630	15.4%	95.1%	93.7%	95.2%	90.8%
3	437	4192	10.4%	91.6%	84.9%	93.3%	80.2%
4	321	6939	4.6%	93.8%	76.7%	94.6%	65.1%
5	723	10,382	7.0%	86.5%	70.2%	84.1%	57.6%
6^a^	923	9269	10.0%	83.1%	72.9%	83.1%	65.0%

^a^The results of test 1 of the standard flow cytometer are shown in Fig. 6a. The results of test 1, test 2 and test 6 of the standard flow cytometer are shown in Fig. S9 and Table S1.

### Cell sorting

HeLa cells are sorted to test the ability of the sorter to enrich biological samples and investigate the cell viability change before and after sorting. GFP-expressing and RFP-expressing HeLa cells served as target cells and nontarget cells, respectively. The results are shown in Table 5. In terms of purity, the results are close to those of the microbeads experiment. However, there is a certain gap in the recovery rate between the sorting of cells and the sorting of beads. This is because the fluorescence intensity of GFP-expressing HeLa cells is distributed over a much wider range than that of green fluorescent beads. The detection noise level of our homemade system is worse than that in the commercial system. Consequently, GFP-HeLa cells with weak signal intensities cannot be effectively recognized in our system, leading to the highest recovery rate of approximately 60%.

**Table 5:** Cell sorting experiments

Test				Theoretical		Experimental	
	Target cells throughput (eps)	Total cells throughput (eps)	Presorting target cell fraction	Recovery rate	Purity	Recovery rate	Purity
1	270	874	30.9%	94.7%	97.1%	59.6%	91.8%
2	253	1044	24.2%	95.0%	96.2%	61.4%	89.3%
3	353	1508	23.4%	93.2%	94.6%	56.4%	87.8%
4	502	1621	31.0%	90.5%	94.8%	50.0%	82.0%

**Fig. 6:**
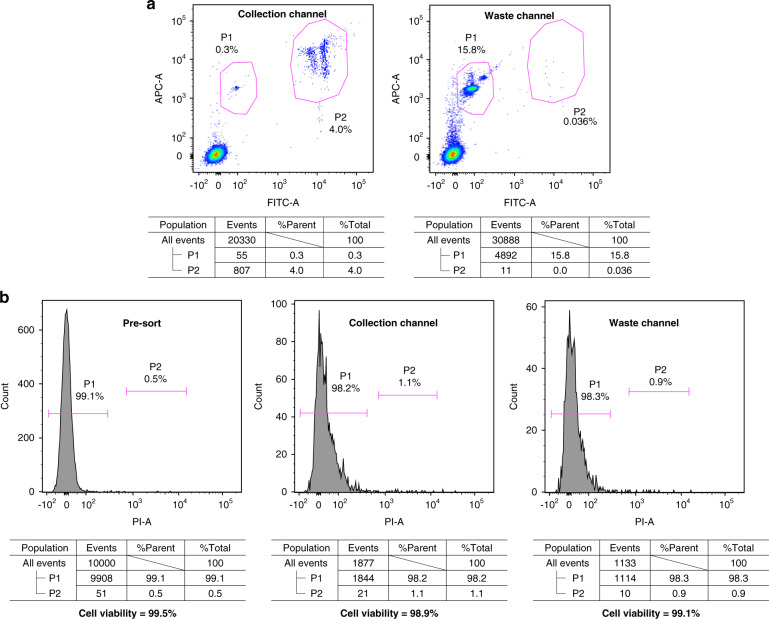
Flow cytometry analysis for sorting outcome and cell viability. **a** Flow cytometry analyses of the outcome of the collection channel and waste channel of the on-chip cell sorter. (Test 1 in Table 4) P1 and P2 represent red beads and green beads, respectively. **b** Flow cytometry analyses of cell viability. (Test 1 in Table 6) To measure viability, HeLa cells were incubated on ice with PI (propidium iodide) solution (500 μg/mL, RuiTaiBio, China) at 4 μL/mL for 5 min. Dead cells stain positively, and thus, viability can be measured by counting the percentage of cells that stain negatively. P1 and P2 represent live and dead cells, respectively.

Cell viability was measured to evaluate the impact of spark-cavitation-bubble-based sorting on cells. As shown in Table 6 and Fig. 6b, cell viability varied slightly after sorting. Additionally, there is little difference in cell viability between the collection channel and waste channel, which indicates that the whole process from spark discharge to jet flow deflection has a negligible effect on cell viability.

**Table 6:** Viability of HeLa cells before and after sorting.

Test	Viability before sorting	Viability of cells in collection channel	Viability of cells in waste channel
1^a^	99.5%	98.9%	99.1%
2	99.7%	99.1%	99.2%
3	99.7%	99.2%	99.3%

^a^The test results for test 1 of the standard flow cytometer are shown in Fig. 6b.

## Discussion

Researchers believe that one of the major advantages of on-chip cell sorting is that it can solve the cross-contamination problem by using disposable microfluidic chips. Unfortunately, most of these sorters require precise and sophisticated fabrication and integration methods, which make the chips unlikely to be replaced frequently due to cost issues. For our sorter, the low fabrication cost (<20 USD) and sample structure make it possible to achieve the mass production of disposable chips, which is helpful to fully realize the advantage of on-chip sorting.

In this work, the microchannels that complete the 3D hydrodynamic focusing are developed in our former work^27^. Since our sorter pushes the entire flow segment containing the target cell into the collection channel, the sorting force (the cavitation bubble size) should be adjusted according to the channel size. Intuitively, a larger channel size requires a stronger force or a larger cavitation bubble, and a smaller channel requires a smaller cavitation bubble.

The sorter can perform fast and accurate sorting on microbeads. The recovery rate in the cell sorting experiment is not as good as expected because of the high noise level of the system. Further work should be performed to improve the system signal-to-noise ratio to realize a better recovery rate for biological samples. Traditional jet-in-air sorting can achieve multichannel sorting by adjusting the electric charge on the cells. For on-chip FACS, considering system complexity, most of the designs are limited to two channels. In the present design of the spark-cavitation bubble cell sorter, only the jet flow during the expansion phase of the cavitation bubble is utilized to deflect cells. Using the reversed jet flow created by the shrinking of cavitation bubbles to achieve multichannel sorting is one of the goals of our future research. Another approach is to build multiple collection channels and to generate different-size cavitation bubbles (corresponding to different force magnitudes) to push different cells into their corresponding collection channels. In addition, sorting in purity mode, sorting based on spectrum and imaging information will also be studied in the future.

## Conclusion

In this work, a spark-cavitation bubble-based on-chip fluorescence-activated cell sorter is developed. Compared with the current on-chip FACS systems, this sorting mechanism simultaneously combines the merits of rapid switching, simple structure, easy setup and long lifetime. We believe that the spark cavitation bubble sorter has the potential to be a good substitute for commercial instruments.

## Supplementary information

Supplementary material with changes marked up

Video of sorting three microbeads

## References

[1] Fu AY, Spence C, Scherer A, Arnold FH, Quake SR (1999). A microfabricated fluorescence-activated cell sorter. Nat. Biotechnol..

[2] Dittrich PS, Schwille P (2003). An integrated microfluidic system for reaction, high-sensitivity detection, and sorting of fluorescent cells and particles. Anal. Chem..

[3] Huh D (2003). Reversible switching of high-speed air-liquid two-phase flows using electrowetting-assisted flow-pattern change. J. Am. Chem. Soc..

[4] Ho CT, Lin RZ, Chang HY, Liu CH (2005). Micromachined electrochemical T-switches for cell sorting applications. Lab Chip.

[5] Kim HJ, Moon HS, Kwak BS, Jung HI (2011). Microfluidic device to separate micro-beads with various fluorescence intensities. Sens. Actuator B-Chem..

[6] Meineke G, Hermans M, Klos J, Lenenbach A, Noll R (2016). Microfluidic opto-caloric switch for sorting of particles with 3D hydrodynamic focusing based on SLE fabrication capabilities. Lab Chip.

[7] Perroud TD (2008). Microfluidic-based cell sorting of Francisella tularensis infected macrophages using optical forces. Anal. Chem..

[8] Wang XL (2011). Enhanced cell sorting and manipulation with combined optical tweezer and microfluidic chip technologies. Lab Chip.

[9] Baret JC (2009). Fluorescence-activated droplet sorting (FADS): efficient microfluidic cell sorting based on enzymatic activity. Lab Chip.

[10] Mazutis L (2013). Single-cell analysis and sorting using droplet-based microfluidics. Nat. Protoc..

[11] Jakobsson O, Grenvall C, Nordin M, Evander M, Laurell T (2014). Acoustic actuated fluorescence activated sorting of microparticles. Lab Chip.

[12] Ung, W. L. et al. Enhanced surface acoustic wave cell sorting by 3D microfluidic-chip design. *Lab Chip* **17,** 4059-4069 (2017). 10.1039/c7lc00715a 28994439

[13] Cheng Z, Wu X, Cheng J, Liu P (2017). Microfluidic fluorescence-activated cell sorting (μFACS) chip with integrated piezoelectric actuators for low-cost mammalian cell enrichment. Microfluidics Nanofluidics.

[14] Sakuma, S., Kasai, Y., Hayakawa, T. & Arai, F. On-chip cell sorting by high-speed local-flow control using dual membrane pumps. *Lab Chip* **17** (2017). 10.1039/c7lc00536a 28685786

[15] Nitta N (2018). Intelligent image-activated cell sorting. Cell.

[16] Foster, J. S. et al. Multi-stage cartridge for MEMS particle storing system. U.S., US8993311B2 (2012).

[17] Pritchard RH (2019). Cell sorting actuated by a microfluidic inertial vortex. Lab Chip.

[18] Cai KP, Mankar S, Maslova A, Ajiri T, Yotoriyama T (2020). Amplified piezoelectrically actuated on-chip flow switching for a rapid and stable microfluidic fluorescence activated cell sorter. RSC Adv..

[19] Wu TH, Gao LY, Chen Y, Wei K, Chiou PY (2008). Pulsed laser triggered high speed microfluidic switch. Appl. Phys. Lett..

[20] Chen Y (2014). Pulsed laser activated cell sorting with three dimensional sheathless inertial focusing. Small.

[21] Hong ZY (2019). High-speed micro-particle manipulation in a microfluidic chip by directional femtosecond laser impulse. Sens. Actuator A-Phys..

[22] Chen CC, Wang JS, Solgaard O (2006). Micromachined bubble-jet cell sorter with multiple operation modes. Sens. Actuator B-Chem..

[23] De WK (2017). Micro vapor bubble jet flow for safe and high-rate fluorescence-activated cell sorting. Lab Chip.

[24] Chahine, G., Frederick, G., Lambrecht, C., Harris, G. & Mair, H. in *SAVIAC Proceedings of the 66th Shock and Vibrations Symposium*, Vol. 1 (1995).

[25] Surdo S, Diaspro A, Duocastella M (2017). Micromixing with spark-generated cavitation bubbles. Microfluidics Nanofluidics.

[26] Zhao J, You Z (2018). Spark‐generated microbubble cell sorter for microfluidic flow cytometry. Cytom. Part A.

[27] Zhao J, You Z (2016). A Microflow Cytometer with a rectangular quasi-flat-top laser spot. Sensors.

[28] Han Y (2021). Diffractive beam shaper for multiwavelength lasers for flow cytometry. Cytom. Part A.

[29] Eubank PT, Patel MR, Barrufet MA, Bozkurt B (1993). Theoretical-models of the electrical-discharge machining process .3. the variable mass, cylindrical plasma model. J. Appl. Phys..

[30] Bhattacharyya B, Doloi BN, Sorkhel SK (1999). Experimental investigations into electrochemical discharge machining (ECDM) of non-conductive ceramic materials. J. Mater. Process. Technol..

[31] Jiao ZH, Zhao JJ, Han Y, Chao ZX, You Z (2021). Dynamics of spark cavitation bubbles in a microchamber. Microfluidics Nanofluidics.

[32] Han Y, Jiao Z, Zhao J, Chao Z, You Z (2021). A simple approach to fabricate ayer glass microfluidic chips based on laser processing and thermocompression bonding. Microfluidics Nanofluidics.

[33] Iino T (2019). High-speed microparticle isolation unlimited by Poisson statistics. Lab Chip.

