# Supplementary material for: Rapid switching and durable on-chip spark-cavitation-bubble cell sorter
Source: Microsyst Nanoeng. 2022 May 18;8:52. doi: 10.1038/s41378-022-00382-2 (PMC9117265; doi:10.1038/s41378-022-00382-2)
Supplement: Supplementary file 1 — Supplementary material with changes marked up [file 41378_2022_382_MOESM1_ESM.docx]

**Supplementary Materials**

Zeheng Jiao,^1, 2, 3^ Yong Han,^1, 2, 3^ Jingjing Zhao,^4*^ Zixi Chao,^1, 2, 3^ Attila Tárnok,^2, 5, 6^ Zheng You,^1, 2, 3^*

1 State Key Laboratory of Precision Measurement Technology and Instrument, Tsinghua University, Beijing 100084, China

2 Department of Precision Instrument, Tsinghua University, Beijing 100084, China

3 Beijing Laboratory for Biomedical Detection Technology and Instrument, Tsinghua University, Beijing 100084, China

4 Department of Structural Biology, Stanford University, School of Medicine, Stanford, CA 94305-5126, USA1

5 Institute for Medical Informatics, Statistics and Epidemiology (IMISE), University of Leipzig, Leipzig, Germany

6 Department of Therapy Validation, Fraunhofer Institute for Cell Therapy and Immunology IZI, Leipzig, Germany

*Correspondence to:

Jingjing Zhao:

Department of Structural Biology, Stanford University, School of Medicine, Stanford, CA 94305-5126, USA; Phone: 6503139585

Email: [zhaojj@stanford.edu](about:blank)

Zheng You: Department of Precision Instrument, Tsinghua University, Beijing 100084, China; Phone: 0086-010-62786000

Email: yz-dpi@mail.tsinghua.edu.cn

The first two authors contributed equally to this work.


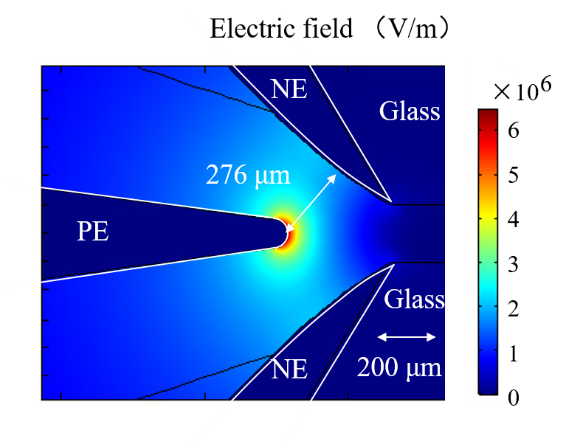


Fig.S1 Electric field derived by finite element analysis, 550 V is applied on the PE. The minimum distance between two electrodes is 276 μm. The electric field in the main channel is about 10,000 V/m, much lower than that in commercial FACS where the cells encapsulated in single droplets are electrostatically deflected.


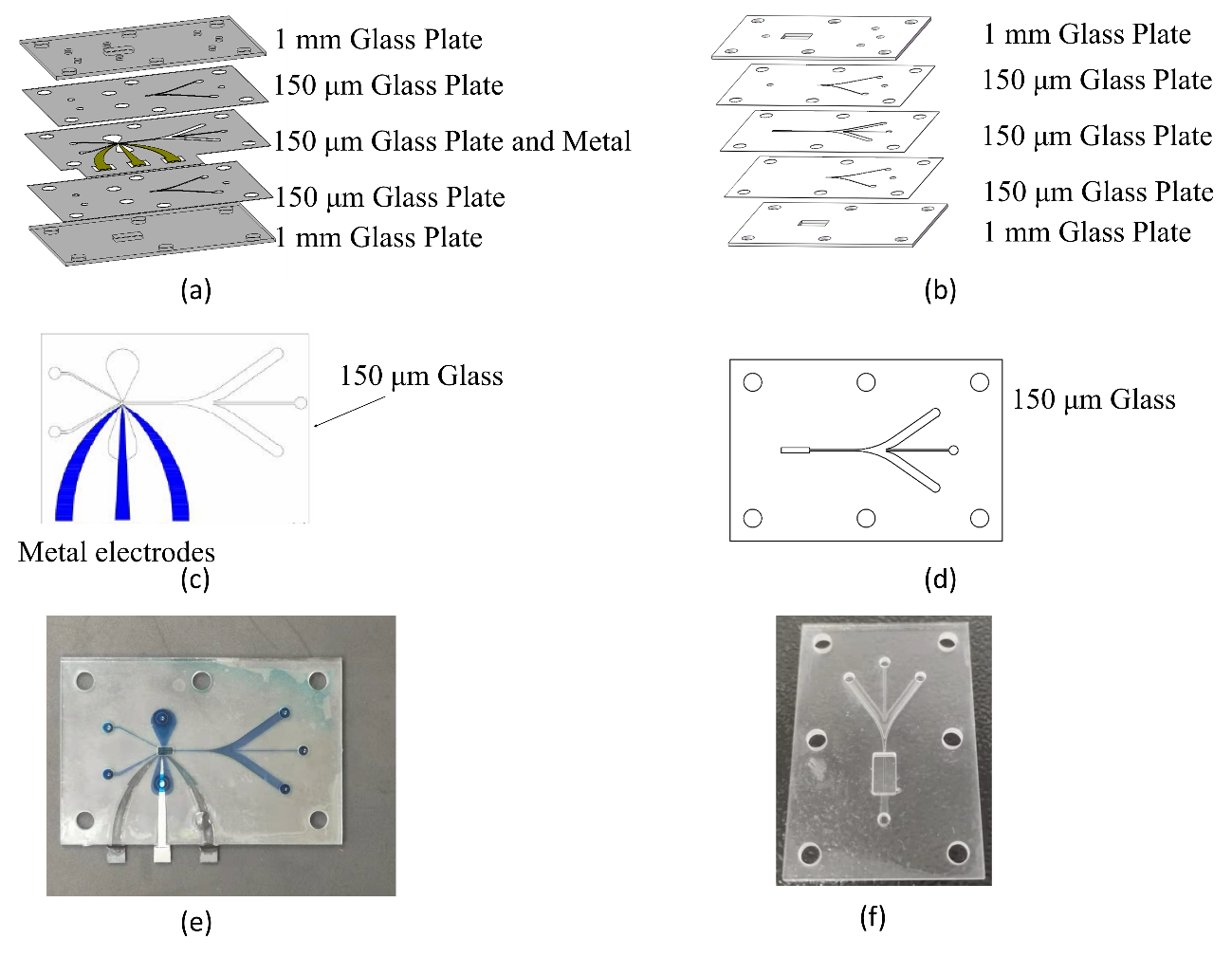


Fig. S2 Comparison of chip between this work and reference [32]. (a) The chip structure with electrodes in this work. (b) The five-layer all-glass chip in reference [31]. The third layer of in this work (c) and reference [32] (d). The microfluidic chips of this work (e) and reference [32] (f).


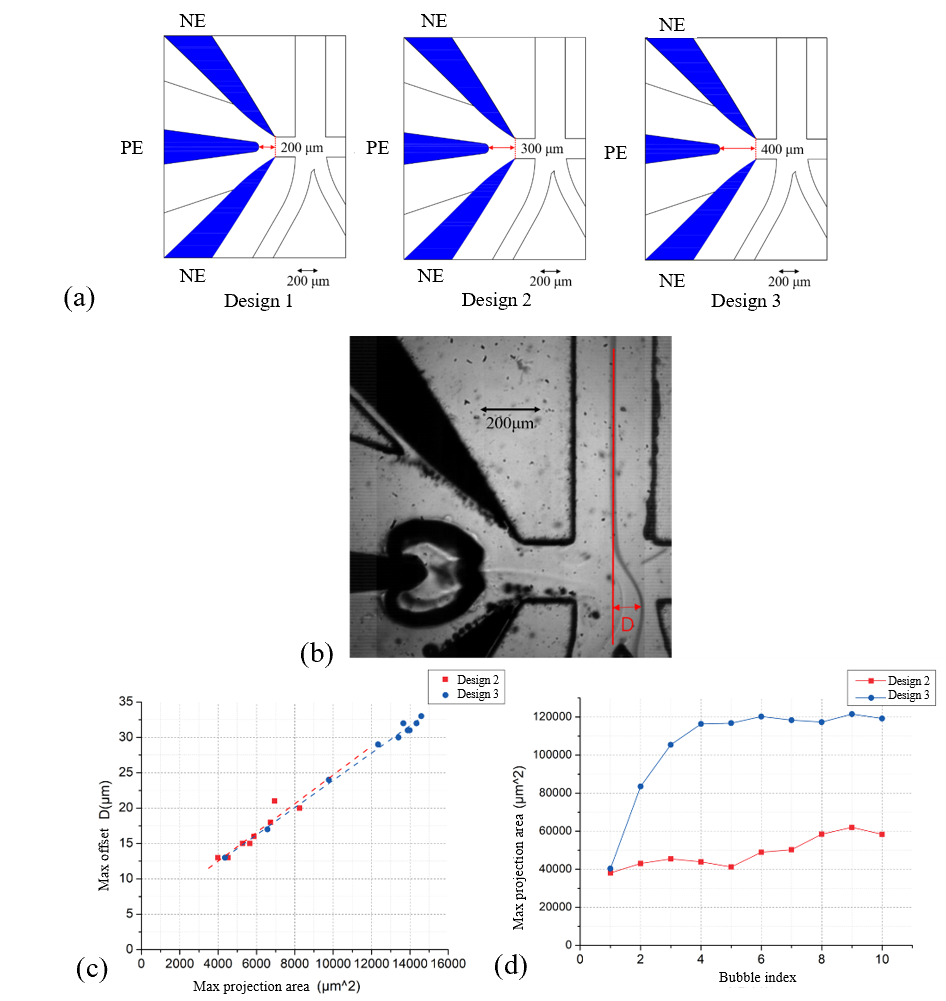


Fig. S3 (a) Three different designs of PE and NE spacing. (b) The sample flow deflection caused by the bubble jet flow. (c) The pushing effects of the different bubble sizes in Design 2 and Design 2. (d) With the buffer flow rate of 10 μL/ s and the action frequency of 200Hz, the bubble size increases with the number of the actions.

**Design of the electrode spacing:**

The design of electrode spacing is determined via experiments.

First, the NEs spacing equals to the nozzle width. The nozzle width mainly takes into account the effect of flow resistance. If the nozzle is too small, the jet cannot eject efficiently to the main channel, reducing the sorting success rate. If the nozzle is too large, the wide range of cells will be affected by the jet flow, reducing the spatial resolution of sorting

Second, the spacing between PE and NEs are more vital for sorting. As shown in Figure S3, we tested three different electrode distances of 200μm, 300μm, and 400 μm (Fig.S3(a)). In Design 1, we found that if the space between the PE and NEs is too small, it will limit the expansion of cavitation bubbles. Thus, the generated cavitation bubble will be not large enough to give a jet flow of sufficient force to push the target cell. In Design 2 and Design 3, two factors are compared: the driving force and the stability of repeated actions. Fig.S3(b) shows the deflected trajectory of the sample flow (brilliant blue solution) during the action of sorter. The max offset of the sample flow is used to characterize the pushing effect. For the experiments shown in Fig. S3(c), the pushing effect of the bubble is approximately linear with the projection area of the bubble. Namely, the pushing effect are positively associated with the bubble size. Next, we compared the stability of repeated actions under same buffer flow rate (10 μL/ s) at the frequency of 200Hz in Fig. S3(d). Compared with Design 3, Design 2 shows a better stability of the maximum projected area over the successive bubbles, because its distance between PE and NE is smaller and the flushing effect of the buffer flow is more efficient to keep the environment constant. In summary, Design 2 is selected as the final.


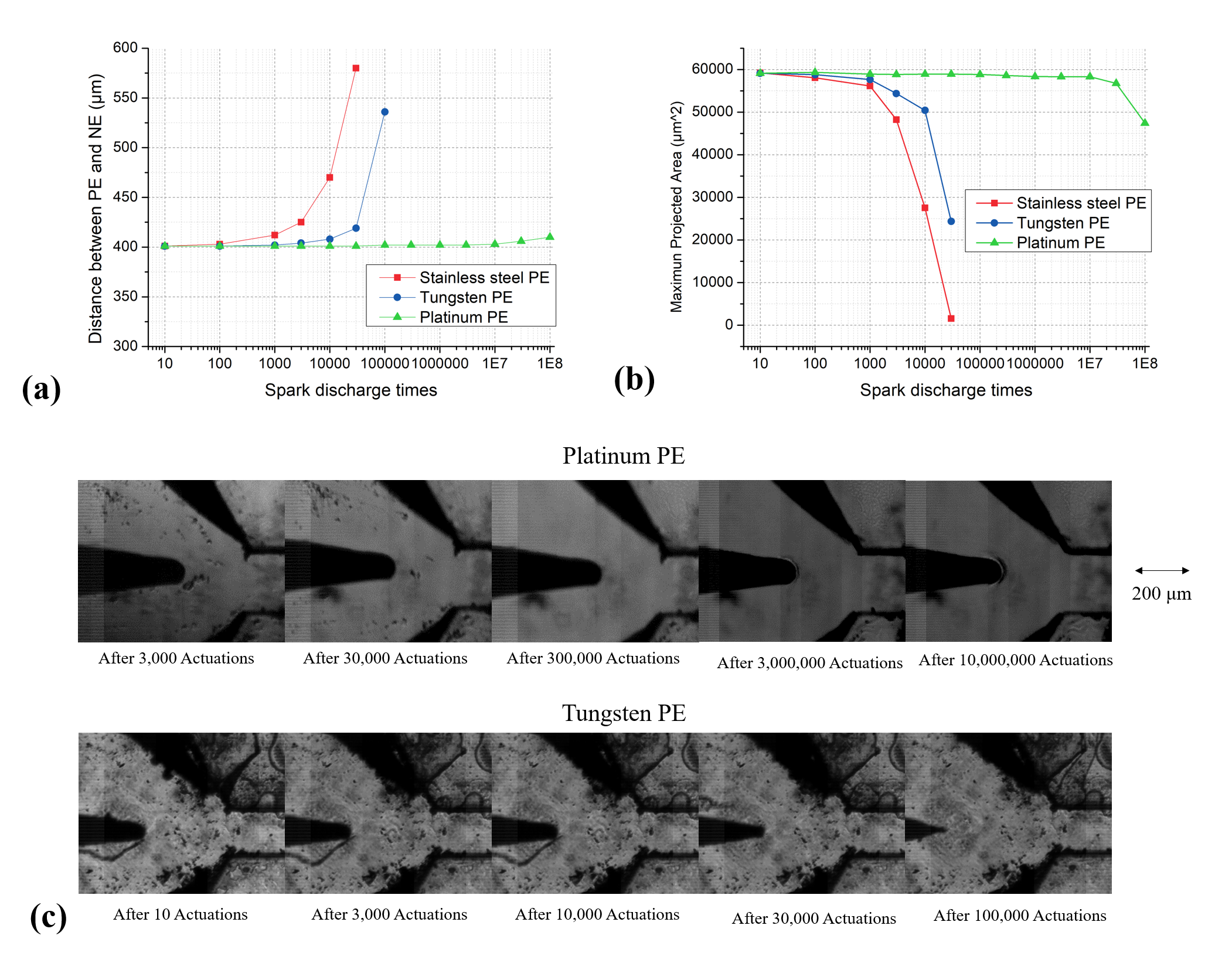


Fig.S4 (a) The distance between the tip of positive electrode (PE) and the tip of negative electrode (NE) after numbers of spark discharges. (b) The maximum projected area of the spark-cavitation bubbles after numbers of spark discharges. The spark discharge is accompanied by the erosion of the PE. For stainless steel PE and tungsten PE, the tip will melt because of the energy deposited after certain times of spark discharge. The gap between PE and NE increases gradually, the breakdown resistance between PE and NE increases, and the cavitation bubbles become smaller and smaller. As for Platinum PE, no obvious erosion can be found after 10^7^ spark discharges. (c) Image of the platinum PE and tungsten PE after numbers of spark discharges.


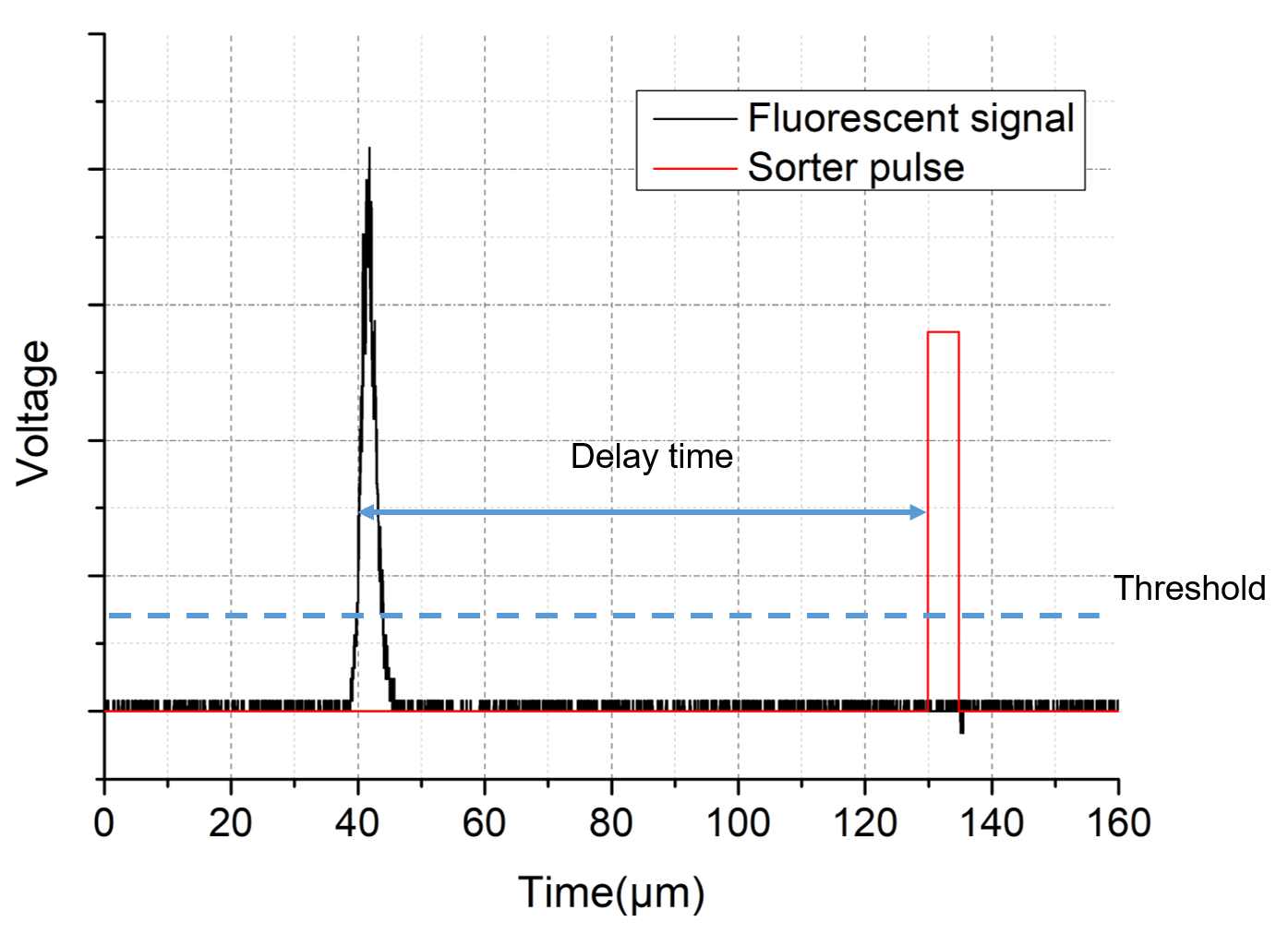


Fig.S5 Timing diagram of a sorting process. A sorting command will be passed to the high-voltage spark generator after a certain delay if the PMT signal surpass the threshold value, then the spark discharge starts immediately to generate a cavitation bubble.


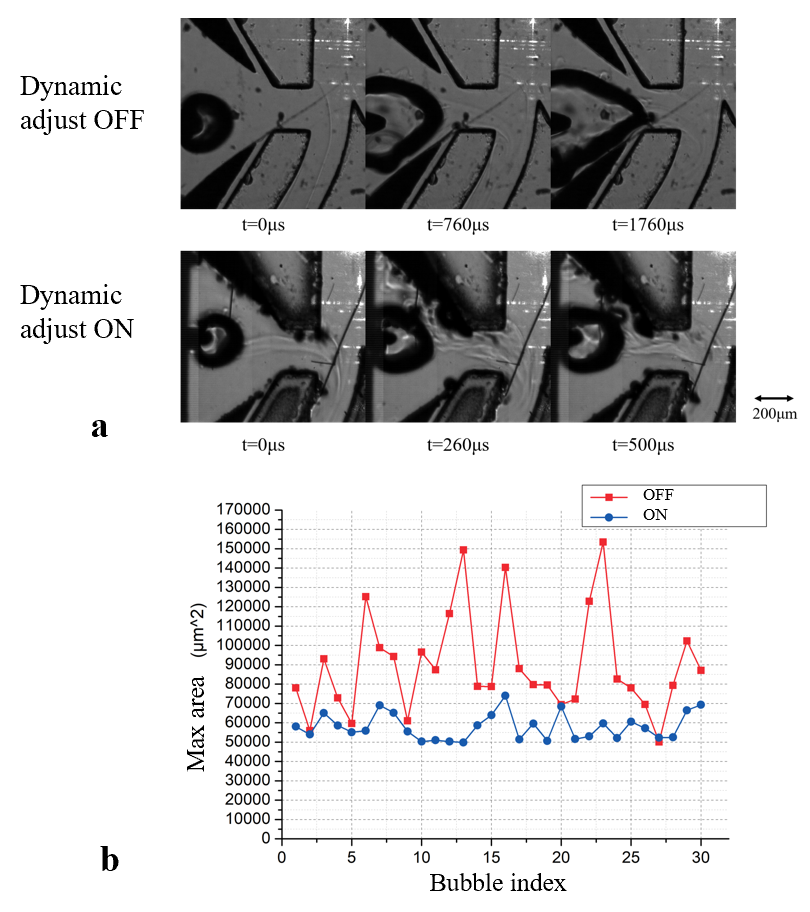


Fig. S6. The changes of the maximum projected area of 30 consecutive cavitation bubble when the algorithm of dynamical adjusting the discharge time is on and off.


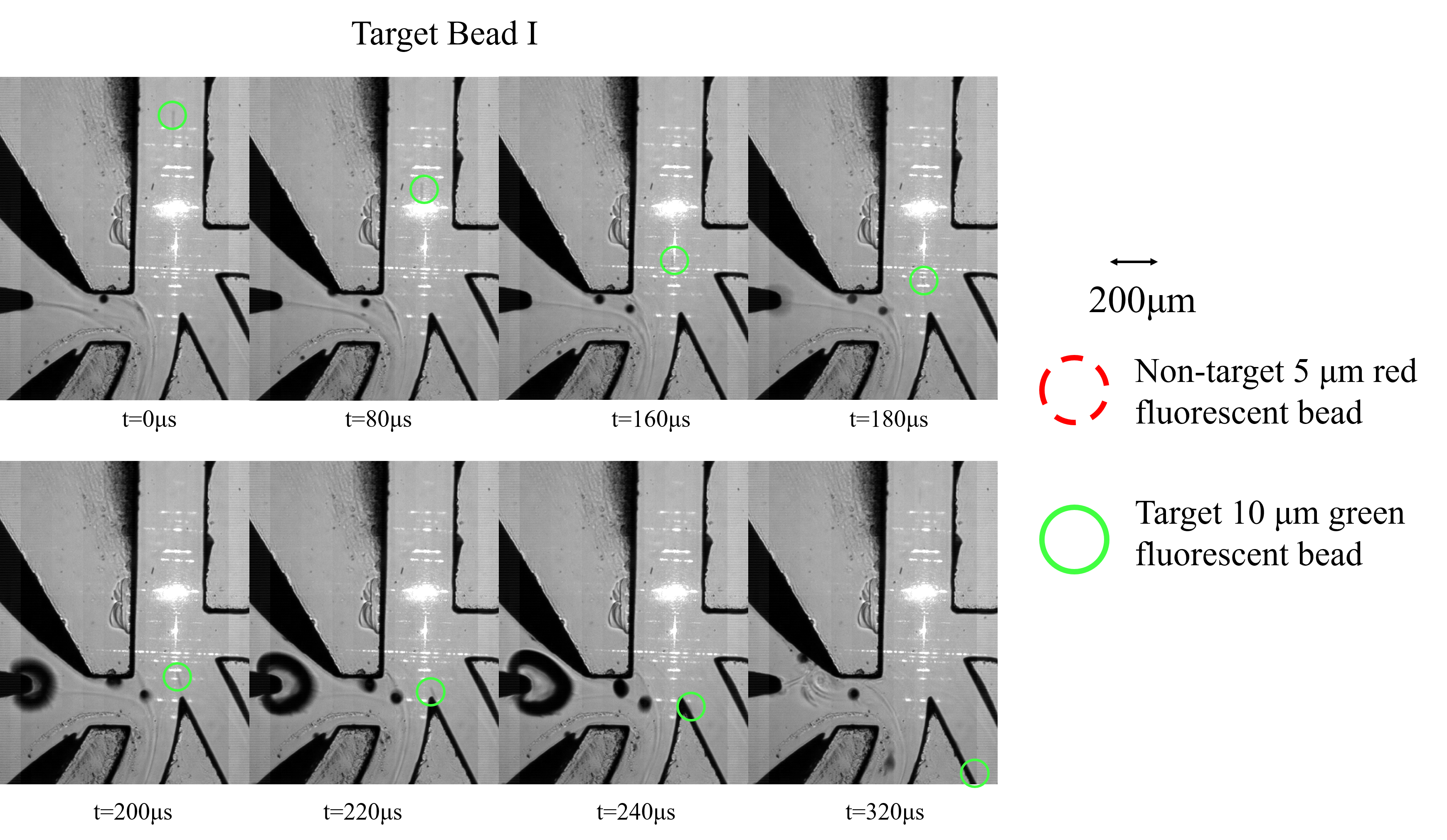

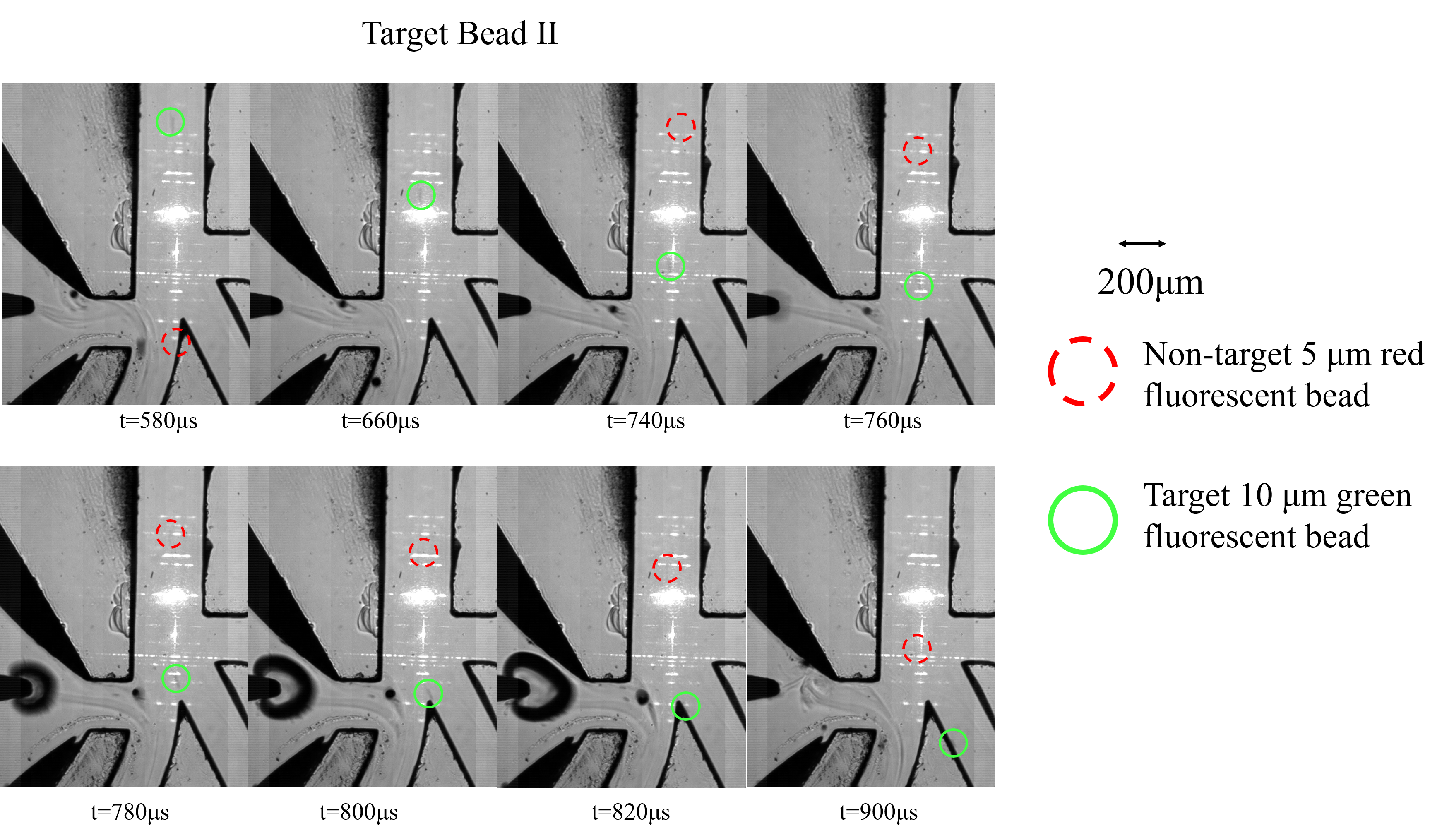

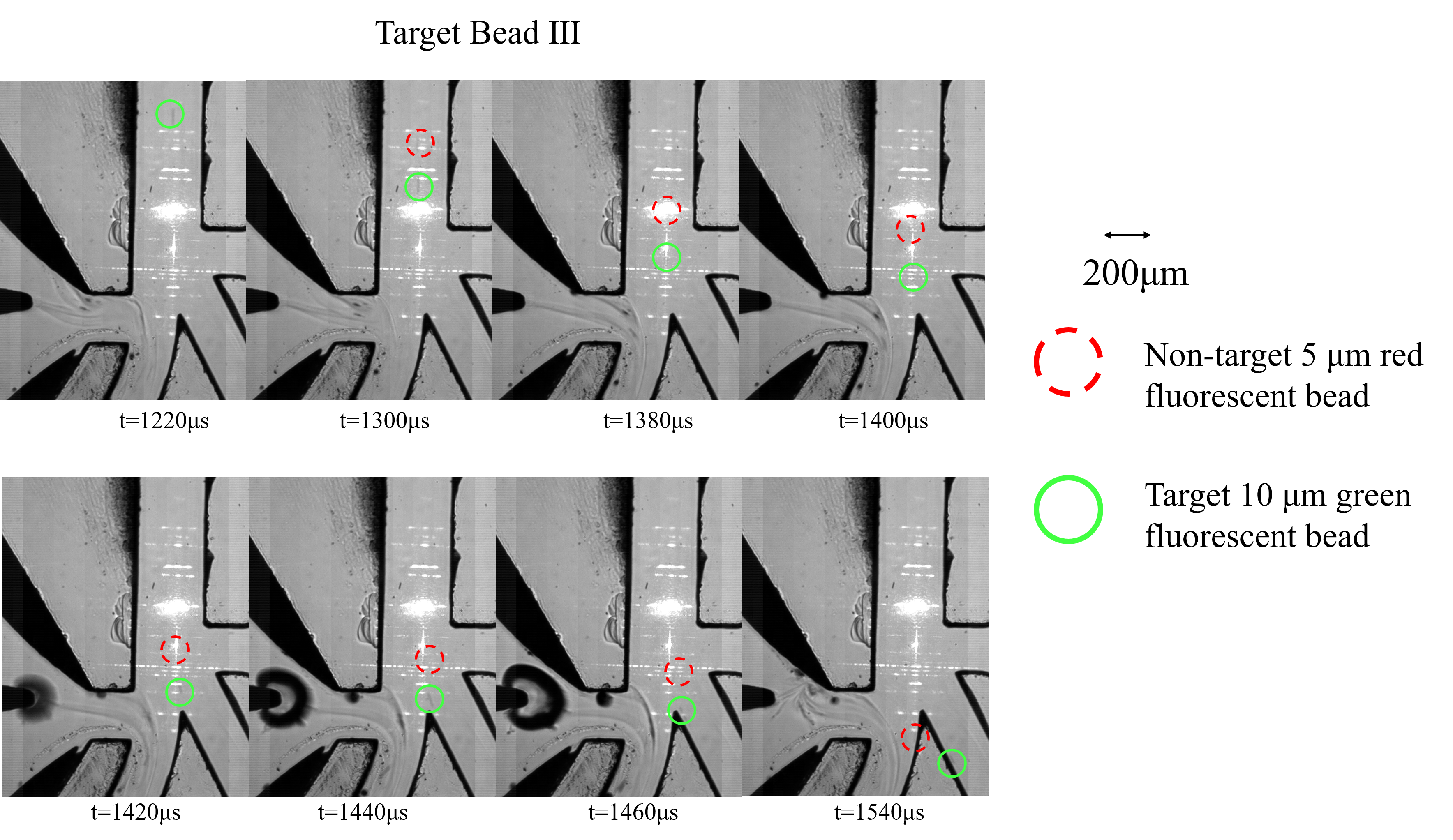


Fig.S7 Three microbeads successively enter the sorting aera and are deflected into the collection channel by three triggered spark cavitation bubbles. The horizontal and vertical positions of the target beads are basically the same when the sorting is triggered, proving the stable performance of the hydrodynamic focusing. The three cavitation bubbles have similar sizes and lifetimes, indicating the efficiency of the algorithm for dynamical adjusting the discharge time. The two non-target beads do not activate sorting and flow into the waste channel.


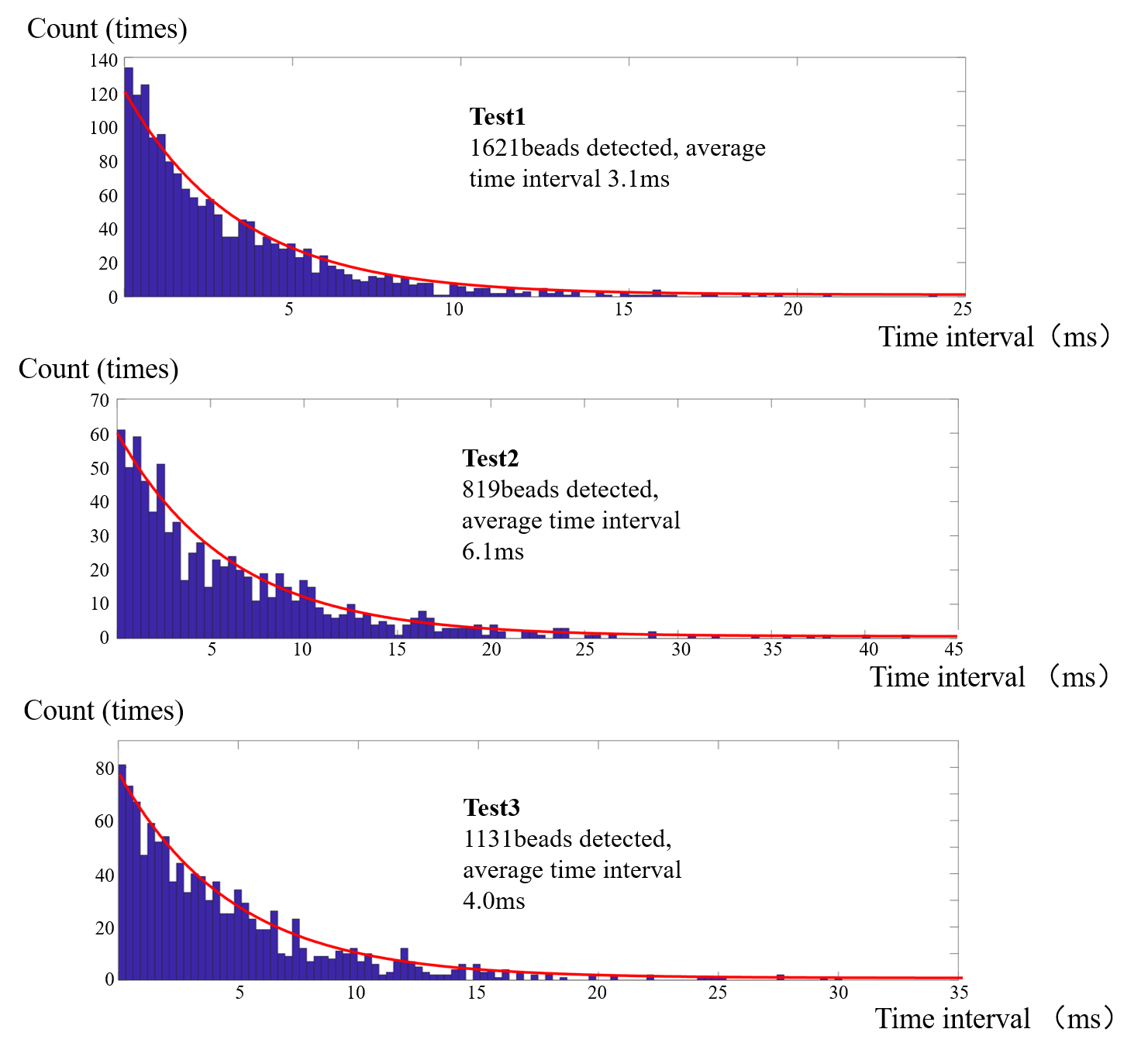


Fig.S8 The histogram of time interval of three groups of beads. The results agree with the probability density function of Poisson distribution (red curves). Namely, it verifies Poisson distribution of time interval of particles.


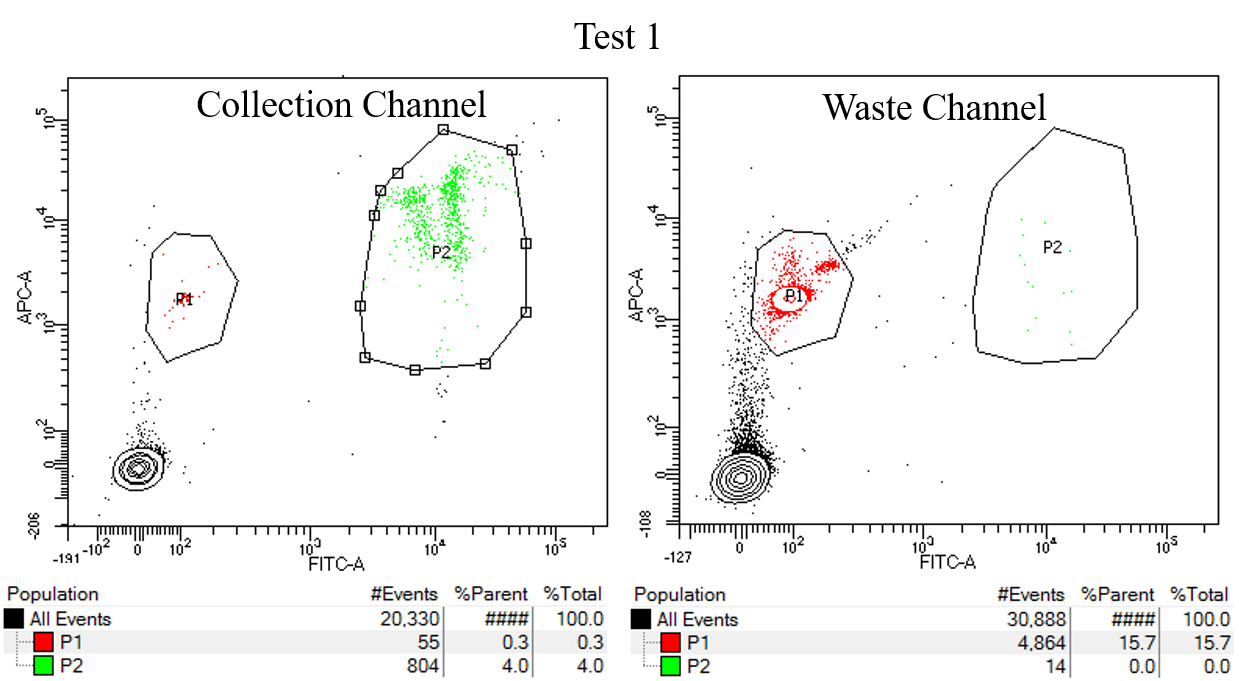

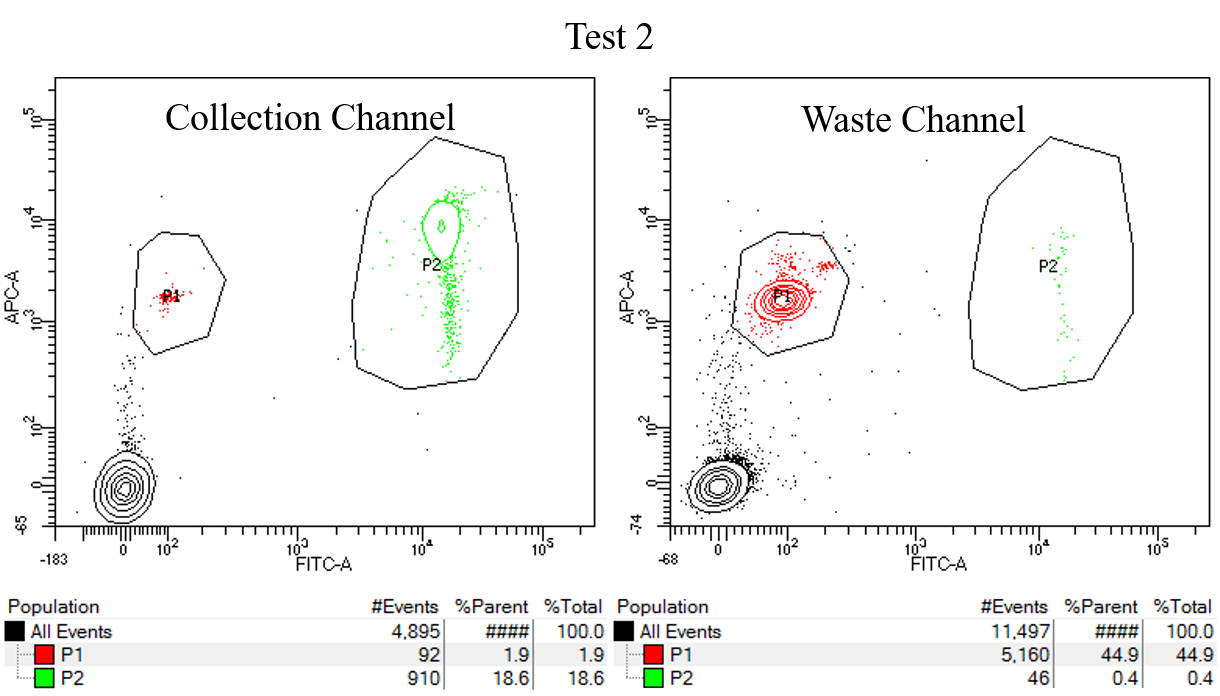

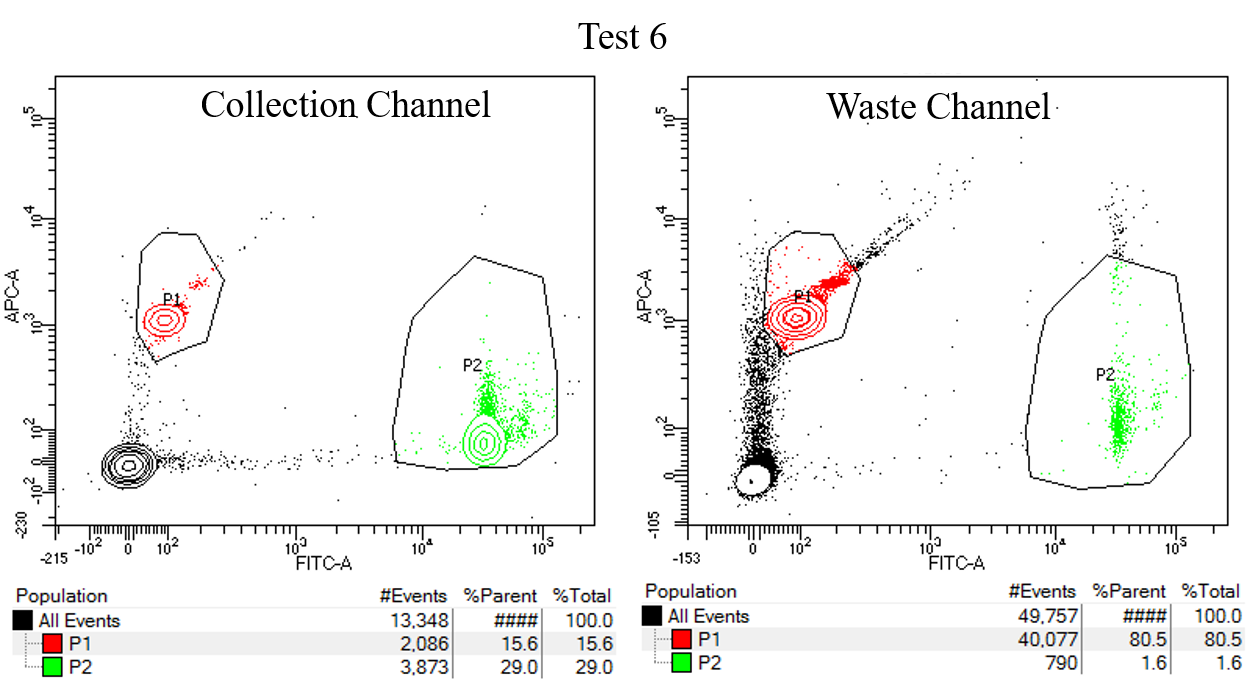


Fig.S9 Flow cytometry analyses of the outcomes of collection channel and waste channel in the on-chip cell sorter. P1 and P2 represent red beads and green beads, respectively.


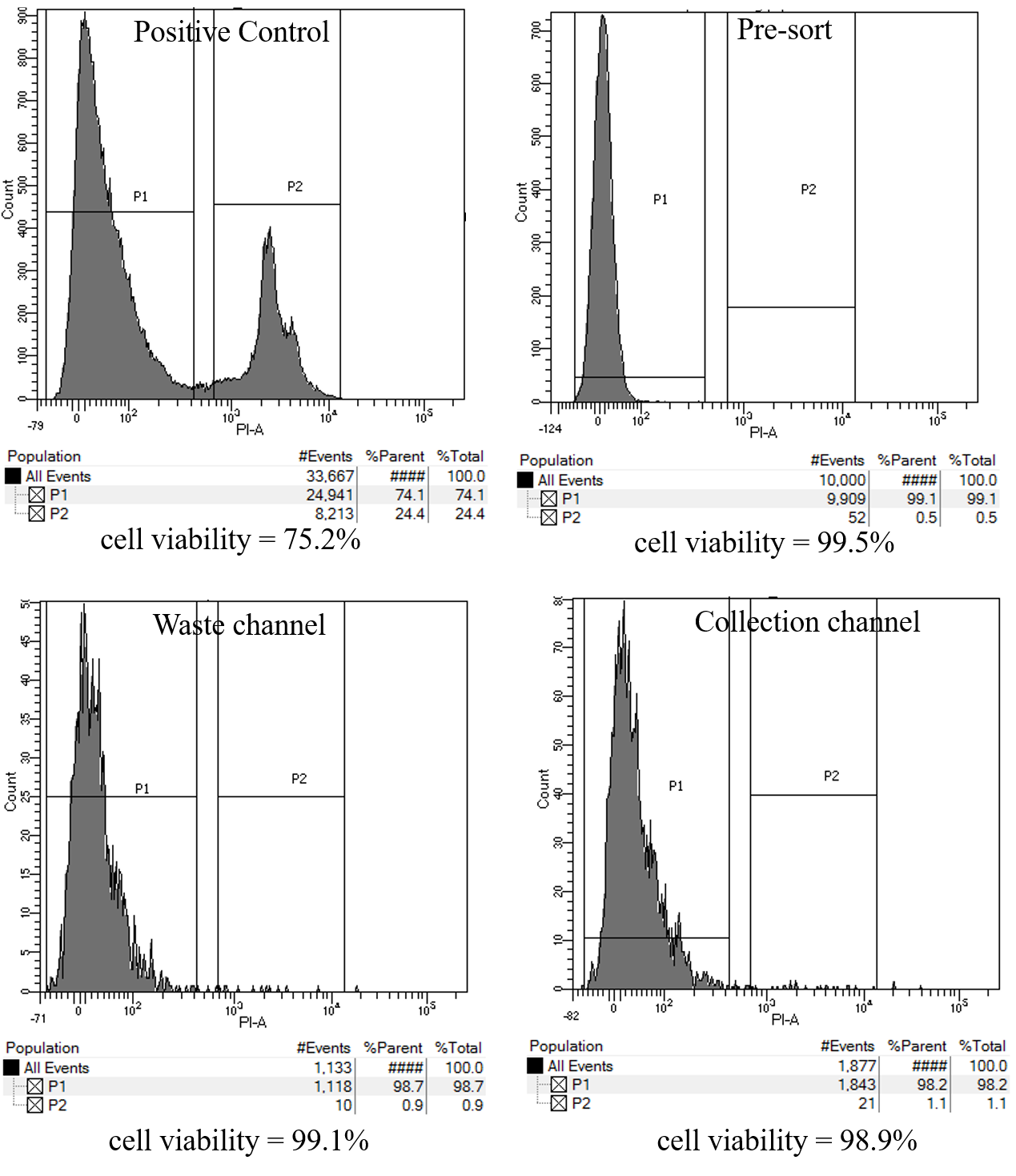


Fig.S10 Flow cytometry analyses of the cell viability. To measure the viability, HeLa cells are incubated on ice with Propidium Iodide (PI) solution (500 μg/mL, RuiTaiBio, China) at 4 μL/mL for 5 minutes, and dead cells are stained positively. The viability is measured by counting the percentage of negatively stained cells. P1 and P2 represent live cells and dead cells, respectively. Samples of hydrogen peroxide induced cell death are served as positive control to locate P2.


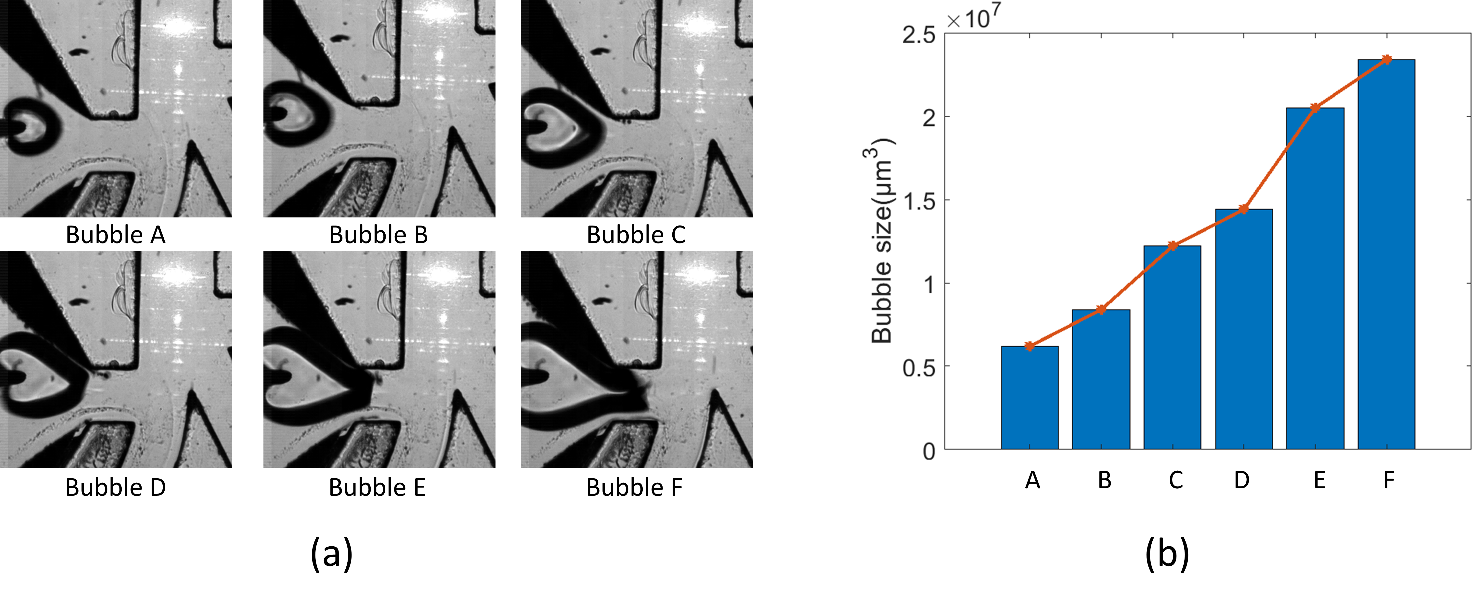


Fig.S11 (a) The images when the six bubbles A-F reaches their maximum volume. (b) Plot of the bubble volumes.

Fig.S11 (a) shows the maximum volumes of the six bubbles. These six energy costs correspond to different bubble sizes, from too small to push the cell (Bubble A), to almost filling the entire cavity (Bubble F). The bubble volume varied linearly, as shown in Fig S11 (b)). Bubble C is chosen on the consideration of a large sorting envelope and a relatively small energy cost. If the bubble size is too large, it takes a long time for the flows to recover and get ready for the next sorting; if too small, the bubble-generated jet flow is insufficient for a successful sorting action.

Table S1. The beads in collection channel and waste channel are used to calculate pre-sorting target beads fraction, the experimental purity, and recovery rate.

| Test | Collection Channel | | Waste Channel | | Total | |  | Experimental | |
| --- | --- | --- | --- | --- | --- | --- | --- | --- | --- |
|  | Green beads | Red beads | Green beads | Red beads | Green beads | Red beads | Pre-sorting target bead fraction | Purity | Recovery rate |
| 1 | 804 | 55 | 14 | 4,861 | 4,916 | 818 | 14.3% | 98.3% | 93.6% |
| 2 | 910 | 92 | 46 | 5,160 | 5,252 | 956 | 15.4% | 95.2% | 90.8% |
| 6 | 3,873 | 2,086 | 790 | 40,077 | 42,163 | 4,663 | 9.96% | 65.0 % | 83.1% |
